# Quantification de la Charge Virale et tests de résistance du VIH-1 aux ARV à partir d’échantillons DBS (Dried Blood Spots) chez des patients Guinéens sous traitement antirétroviral

**DOI:** 10.4102/ajlm.v4i1.168

**Published:** 2015-06-26

**Authors:** Nestor Bangoura, Abou A.M. Diouara, Mohamed Cissé, Halimatou D. Ndiaye, Souleymame Mboup, Ahidjo Ayouba, Coumba T. Kane

**Affiliations:** 1 Service de Dermatologie CHU Donka, CTA, Conakry, Guinée; 2 Laboratoire de Bactériologie Virologie CHU Aristide Le Dantec, Université Cheikh Anta Diop de Dakar, Sénégal; 3 UMI 233 IRD de Montpellier, France

## Abstract

**Problématique:**

Comme dans plusieurs pays du Sud, le suivi virologique des patients sous traitement antirétroviral (TARV) en Guinée est timide voire inexistant dans certaines localités. Le but de cette étude était d’évaluer la faisabilité technique et logistique de l’utilisation des DBS dans les tests de charge virale (CV) et de génotypage.

**Méthode:**

De septembre à octobre 2010, les DBS ont été préparés à partir de prélèvements sanguins de patients adultes sous TARV. Le délai d’envoi des échantillons au laboratoire de référence était de 30 jours maximum après le prélèvement et se faisait à température ambiante. La CV a été quantifiée et les échantillons de patients en échec virologique (CV ≥ 3 log_10_ copies/mL) ont été génotypés selon le protocole de l’ANRS. L’algorithme de Stanford version 6.0.8 a été utilisé pour l’analyse et l’interprétation des mutations de résistance.

**Résultats:**

Parmi les 136 patients inclus, 129 et 7 étaient respectivement sous première et deuxième ligne de traitement avec une médiane de suivi de 35 mois [IQR: 6-108]. L’échec virologique a été noté chez 33 patients. Parmi eux, 84.8% (*n* = 28/33) ont bénéficié d’un génotypage. Le taux de résistance global était de 14% (*n* = 19/136). Le CRF02_AG était le sous type viral le plus prévalent (82%; *n* = 23)

**Conclusion:**

En plus de montrer la faisabilité technique et logistique des tests de CV et de génotypage à partir des DBS, ces résultats montrent l’intérêt de leurs utilisations dans le suivi virologique des patients sous TARV. Cette étude a permis également de documenter l’échec virologique, la résistance aux ARV et la diversité génétique du VIH-1 en Guinée.

**Mots clés:**

VIH-1, Résistance aux ARV, DBS (Dried Blood Spots), Guinée Conakry, Génotypage, Charge Virale.

## Abstract (en)

Quantification of Viral load and resistance tests of HIV-1 to ARVs from dried blood spots samples in Guinean patients undergoing antiretroviral treatment.

**Problem:**

As in several countries of the South, the virological monitoring of patients undergoing antiretroviral treatment (ARVT) in Guinea is low or non-existent in some locations. The aim of this study was to assess the technical and logistical feasibility of the use of (dried blood spots) DBSs in viral load (VL) and genotyping tests.

**Method:**

From September 2010 to October 2010, DBS were prepared from blood samples of adult patients under ARVT. The samples had to be sent to the reference laboratory within 30 days after the sample had been done at ambient temperature. The VL was quantified and the samples of patients with virological failure (CV ≥ 3 log_10_ copies/mL) were genotyped according to the ANRS protocol. The Stanford algorithm, version 6.0.8, was used to analyse and interpret the resistance mutations.

**Results:**

Amongst the 136 included patients, 129 and 7 were under first and second line treatment respectively, and monitored for an average of 35 months [IQR: 6-108]. Virological failure was noticed among 33 patients. Among them, 84.8% (*n* = 28/33) benefited from genotyping. The global resistance rate was 14% (*n* = 19/136). CRF02_AG was the most prevalent viral subtype (82%; *n* = 23)

**Conclusion:**

In addition to demonstrating the technical and logistic feasibility of VL and genotyping tests from DBSs, these results show the relevance of their use in the virological monitoring of patients under ARVT. Also, this study made it possible to provide information on virological failure, ARV resistance and the HIV-1 genetic diversity in Guinea.

## Introduction

En Guinée, la prévalence du VIH chez les adultes (15-49ans) est estimée à 1.4%.^1^ La mise sous traitement antirétroviral (TARV) des patients vivant avec le VIH/SIDA a débuté en 1999. Depuis, le nombre de sites de prise en charge (PEC) opérationnels des patients répartis dans le pays a augmenté et est passé de 46 en 2012 à 51 en fin 2013. La couverture nationale en centres de Conseils et dépistage volontaire (CDV) était de 66 sites et 131 sites de prévention de la transmission mère-enfant (PTME) pour 464 structures offrant les services de consultations prénatales (CPN) en 2013.^2^ De plus, la couverture en TARV a connu une hausse au cours de ces années et est passée de 22.50% en 2007 à 56.91% en fin 2011, pour 40 258 personnes éligibles au TARV selon les lignes directrices de l’OMS.^3^ A l’image de plusieurs pays à ressources limitées, du fait d’un manque d’infrastructures, d’équipements biomédicaux et de personnels qualifiés, les tests moléculaires, notamment la charge virale (CV) et le génotypage, ne sont pas tout le temps disponibles. Par conséquent, le suivi des patients sous TARV en Guinée se fait essentiellement en se basant sur des critères clinico-immunologiques.^4^ L’utilisation du papier buvard comme support de prélèvement sanguin alternatif au plasma permettrait la collecte et le transport des échantillons des sites périphériques vers le centre de référence national ou international. Le papier buvard (DBS) a été largement utilisé dans le diagnostic sérologique et moléculaire de l’infection à VIH.^5,6^ De plus, les DBS ont été utilisés dans la détermination de la CV et des tests de résistance du VIH-1 aux ARV.^7,8,9^ Plusieurs études ont évalué et validé le DBS en le comparant au plasma^10,11,12,13^, qui est le type d’échantillon de référence pour les tests de CV et de génotypage. Le DBS est un support facile d’utilisation, moins exigeant que le plasma et le sérum car ne nécessitant pas une chaine de froid pour la conservation, le stockage et le transport des échantillons. C’est dans ce contexte que s’inscrit cette étude pionnière en Guinée dont l’objectif était d’évaluer la faisabilité technique et logistique des tests de CV et de génotypage du VIH-1 à partir d’échantillons DBS de patients sous TARV collectés dans des conditions de terrain.

## Conception et méthode d’étude

### Patients et sites de collecte des échantillons

**FIGURE 1:**
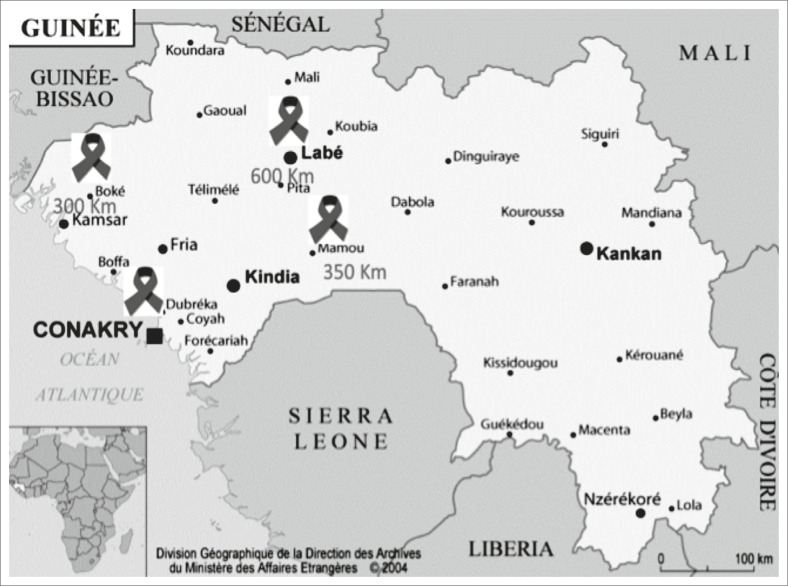
Répartition des sites de collecte des échantillons.

Cette étude a porté sur des patients adultes (≥ 18 ans) sous TARV depuis au moins 6 mois, suivis dans le cadre du programme national. Les patients inclus dans cette étude ont été recrutés consécutivement sur la période allant de septembre à octobre 2010 au niveau de 4 sites de prise en charge (PEC). Les individus infectés par le VIH-2 ou coinfectés par le VIH-1 et VIH-2, de même que les femmes ayant bénéficiés d’une prévention de la transmission mère-enfant du VIH (PTME), n’étaient pas inclus. Le choix de ces sites a été fait de façon aléatoire et sur la base de l’existence d’association de personnes vivant avec le VIH pouvant faciliter la collecte des échantillons. Un des sites est situé dans la capitale, le Centre de Traitement Ambulatoire de Conakry, et les 3 autres sont localisés dans les régions de Boké, Mamou et Labé, distants respectivement de 300, 350 et 600 km de la capitale (Figure 1).

### Echantillonnage et conservation des prélèvements DBS

Pour chaque patient, 5 mL de sang total ont été recueillis dans un tube EDTA par ponction veineuse au niveau du pli du coude pour servir à la préparation de 2 cartes de papier filtre Whatman 903^®^, conformément aux règles d’hygiène et de sécurité , et 50 µL de sang total ont été déposés sur chacun des 5 spots à raison d’une carte DBS, préalablement identifiée et datée. Les échantillons ont été séchés pendant la nuit à température ambiante (30 à 37 °C). Chaque échantillon DBS a été placé dans un sachet plastique individuel hermétiquement fermé en présence de dessiccateurs. Puis, ils ont été conservés et stockés sur site à température ambiante. L’envoi des DBS vers le laboratoire de Bactériologie Virologie de l’Hôpital Aristide Le Dantec de Dakar au Sénégal pour les tests de CV et de génotypage s’est fait par voie terrestre dans les 30 jours suivant le prélèvement.

### L’extraction des acides nucléiques

A partir de 2 spots de chaque échantillon DBS/patients et à l’aide de l’appareil NucliSENS miniMAG (bioMérieux, Craponne, France), les acides nucléiques totaux ont été obtenus par extraction magnétique et manuelle selon la chimie de Boom.^14^ En effet, les spots découpés à l’aide d’un « puncher » dédié ont été trempés dans un tube contenant 2 mL de tampon de lyse et agité pendant 30 minutes à température ambiante. Les acides nucléiques ont été extraits et élués dans 25 µL de tampon d’élution après une série de centrifugation et des lavages avec des tampons 1, 2 et 3 comme précédemment décrit.^10^

### La quantification de la charge virale (CV)

La détermination de la CV a été effectuée à partir de l’extrait obtenu et ceci en utilisant kit NucliSENS EasyQ HIV-1 v2.0 (bioMérieux, Craponne,France) conformément aux instructions du fabriquant. La plateforme utilisée était NucliSENS® EasyQ (BioMérieux, Lyon, France) et le principe est une amplification de type NASBA. Le seuil de détectabilité de la technique est de 800 copies/mL.15 Dans cette présente étude, le seuil de l’échec virologique a été fixé à 3 log_10_ copies/mL

### Génotypage et analyse phylogénétique

Le génotypage a été effectué selon le protocole de l’ANRS (http://www.hivfrenchresistance.org/) qui consiste à faire des amplifications séparées par PCR des fragments de la protéase en entier et des 240 premiers codons de la reverse transcriptase (RT) du gène *pol* en utilisant respectivement les couples d’amorces 5’Prot1/3’Prot1 et MJ3/MJ4 comme amorces externes et 5’Prot2/3’Prot2 et A35/NE35 comme amorces internes. Les produits de PCR de 2^ème^ tour ont été purifiés avec le kit QIAquick Gel Extraction Kit®, (Qiagen, Courtaboeuf, France) conformément aux indications du fabriquant. L’ADN purifié a été directement séquencé sur la plateforme ABI Prism 3100 Avant (Genetic analyzer Applied Biosystem) selon la technologie du Big Dye Terminator Technology®v3.1 (Applied Biosystems, Courtaboeuf, France). Les séquences obtenues ont été assemblées et manuellement éditées sur le logiciel SeqManTM II 5.08 de la suite de DNAstar® software (Lasergene, Konstanz, Germany). L’analyse et l’interprétation des mutations de résistance ont été réalisées sur l’algorithme de l’Université de Stanford version 6.0.8 (http://hivdb.stanford.edu/). Les séquences générées ont été alignées avec des séquences références du VIH-1 groupe M et l’ensemble des formes recombinant les CRFs disponibles sur Los Alamos hiv database (http://www.hiv.lanl.gov/content/index). Trois séquences de chaque sous-type pur ont été incluses dans l’alignement. Et les séquences ont été alignées avec l’algorithme de MUSCLE puis l’alignement dégapé obtenu a été avec le programme de Gblocks du logiciel SEAVIEW v4.4.1. L’arbre phylogénétique de Maximun de vraisemblance (PhyML) a été également généré sur SEAVIEW v4.4.1 avec comme paramètres supports de branche déterminés par la méthode *approximate likelihood ratio test* (aLRT), option SH-like. L’analyse de similarité et de bootscanning pour la confirmation des formes recombinantes (CRFs, URFs) a été effectuée sur le logiciel Simplot v3.5.1.^16,17^ L’analyse et les calculs statistiques des données ont été réalisés avec les logiciels Epi Info v3.5.4 et Microsoft Excel.

## Considérations éthiques

Cette étude est une sous-étude d’un projet multicentrique impliquant 3 pays de l’Afrique de l’Ouest (Sénégal, Mali et la République de Guinée) et a été approuvée par les comités éthiques nationaux de ces pays. Les patients ont été recrutés consécutivement sur base volontaire, sur une période allant de septembre à octobre 2010 après signature d’un formulaire de consentement libre et éclairé. Pour garder confidentielles les données des participants, un code unique a été attribué à chaque prélèvement.

## Résultats

### Caractéristiques de la population d’étude

Au total 136 patients infectés par le VIH-1 sous TARV ont participé à cette étude. Parmi eux, 129 étaient sous traitement de première ligne (2INTI + 1INNRT) et 7 sous deuxième ligne (2INTI + 1IP/r), avec une médiane de suivi thérapeutique de 35 mois [IQR: 6-108 mois]. Le sexe ratio Homme/Femme était de 0,64 et l’âge médian était de 38 ans [IQR: 18-61 ans] Boîte 1.

### Charge virale (CV) et tests de résistance

L’échec virologique (CV ≥ 3 log_10_ copies/mL) a été observé chez 33 patients soit un taux de 24.26% (*n* = 33/136). Parmi eux, 4 étaient sous deuxième ligne de TARV et 13/29 patients ont eu des changements de molécules de première ligne (exemple: d4T par AZT) pour des raisons cliniques ou de tolérance. Dans le Tableau 1, figurent entre autre l’historique du traitement et les données virologiques des patients en échec virologique. Selon la durée du traitement, 4/13, 7/31 et 22/92 patients étaient en échec virologiques respectivement à des intervalles 6-12, 13-24 et > 24 mois.

**TABLEAU 1:** Données liées aux patients en échec virologique (CV ≥ 3 log_10_ copies/mL).

Sites de collectes des DBS	Codes Patients	Traitement ARV antérieur	Traitement ARV en cours	Durée du traitement (mois)	CV (log_10_copies/mL)	Mutations de résistance INRT	Mutations de résistance INNRT
Conakry	GUIN_080	-	AZT+3TC+NVP	26	6.08	M184V,T215ST	V106A,E138K
	GUIN_102	-	AZT+3TC+NVP	30	6.42	M41L,M184V,T215Y	V90I, V108I, Y181C, H221Y
	GUIN_113	-	AZT+3TC+NVP	36	3.42	M41L, D67G, T69AD, K70R, V75M, M184V, T215Y	K101EK, K103N
	GUIN_123	-	AZT+3TC+NVP	42	4.22	M184V, T215F	K101P
	GUIN_125	AZT+3TC+NVP	d4T+3TC+NVP	6	3.62	K70KR	V90I
	GUIN_132	-	AZT+3TC+EFV	25	5.53	-	-
	GUIN_142	-	AZT+3TC+NVP	23	4.36	-	-
	GUIN_176	-	d4T+3TC+NVP	16	3.25	-	K103EK
	GUIN_198	AZT+3TC+NVP	ABC+IDV+ LPVr	108	6.09	D67N, K70R, M184V, L210W, T215Y, K219E	K103N, Y181C, G190A, H221Y
	GUIN_204	AZT+3TC+NVP	AZT+3TC+LPVr	39	5.20	T69N	V179E
	GUIN_207	-	AZT+3TC+NVP	40	6.75	T69N, L74I, Y115F, M184V, K219EK	V90I, K103N, V108I, V179E, P225H
	GUIN_209	d4T+3TC+NVP	AZT+3TC+NVP	33	5.39	-	V90IV, Y181C
	GUIN_213	AZT+3TC+NVP	TDF+ABC+LPVr	36	4.93	-	-
	GUIN_214	AZT+3TC+NVP	d4T+3TC+NVP	7	3.64	-	-
Mamou	GUIN_003	AZT+3TC+EFV	AZT+3TC+NVP	53	4.87	-	-
	GUIN_006	AZT+3TC+EFV	AZT+3TC+LPVr	18	3.68	-	-
	GUIN_009	-	d4T+3TC+NVP	77	3.49	D67N, K70R, M184V, K219Q	K103N
	GUIN_021	-	AZT+3TC+NVP	6	3.91	M41L, D67N, K70R, M184V, T215Y	K103N, V106M
	GUIN_031	AZT-DDI-EFV	AZT+3TC+EFV	77	3.54	D67d, T69G, M184V, T215F, K219E	K103N, Y188H
	GUIN_032	d4T+3TC+NVP	AZT+3TC+NVP	52	3.36	--	-
	GUIN_033	-	d4T+3TC+NVP	70	3.40	-	-
	GUIN_005	AZT+3TC+NVP	d4T+3TC+NVP/IDV	56	3,63	Non Amplifié	Non Amplifié
	GUIN_004	AZT+3TC+NVP	d4T+3TC+NVP	60	3,27	Non Amplifié	Non Amplifié
Boké	GUIN_076	-	AZT+3TC+NVP	44	3.08	D67H, K70R, M184V, K219E	V106I,Y188L
	GUIN_077	-	d4T+3TC+NVP	6	5.11	-	K103T,G190AG
	GUIN_095	AZT+3TC+EFV	d4T+3TC+NVP	21	3.15	M184V	K103N
	GUIN_099	-	d4T+3TC+NVP	17	3.80	--	-
	GUIN_164	AZT+3TC+NVP	d4T+3TC+NVP	21	3,13	Non Amplifié	Non Amplifié
	GUIN_028	-	AZT+3TC+NVP	44	3,07	Non Amplifié	Non Amplifié
	GUIN_206	d4T+3TC+NVP	AZT+3TC+NVP	20	3,06	Non Amplifié	Non Amplifié
Labé	GUIN_048	-	AZT+3TC+EFV	30	3.36	M184V,T215Y	K103N,V108I
	GUIN_050	d4T+3TC+NVP	AZT+3TC+NVP	27	5.81	-	K103KN,G190AG
	GUIN_053	-	d4T+3TC+NVP	86	5.39	T69N, K70R, M184V, K219Q	V108I,Y181C

**BOÎTE 1:** Caractéristiques de la population d’étude et nombres de participants par sites.

Caractères		Effectifs
**Sites de collectes**	Conakry (CHU de DONKA)	44
	Boké (Hôpital régional)	33
	Mamou (Hôpital régional)	35
	Labé (Hôpital régional)	24
	Sexe ratio (Homme/femme)	0.64
	Médiane d’âge	38 [IQR : 18-61 ans]
	Première ligne (2INTI+1INNTI)	129
	Deuxième ligne (2INTI+1IP)	7
	Médiane de suivi thérapeutique	35 [IQR : 6-108 mois]

INRT, inhibiteur nucléosidique de la reverse transcriptase; NNRTI, inhibiteur non nucléosidique de la reverse transcriptase; AZT, zidovudine; 3TC, lamivudine; ABC: abacavir; NVP, nevirapine; EFV, efavirenz; d4T, stavudine; IDV, indinavir; LPV/r, lopinavir/ritonavir.

**FIGURE 2:**
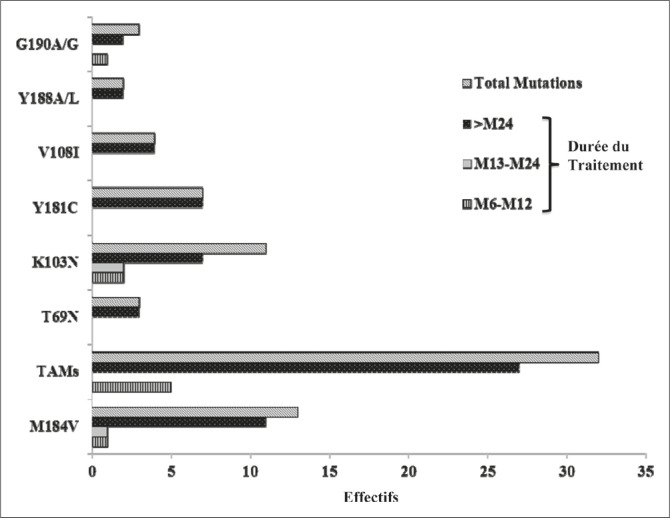
Fréquences des mutations observées en fonction de la durée du Traitement.

Au total, 28/33 échantillons de patients en échec virologique ont pu être génotypés soit un taux d’amplification réussi de 84.84%. La médiane de CV de ces échantillons de patients en échec virologique génotypés (*n* = 28) était de 4 log_10_ copies/mL [IQR: 3-6.7] et celui des échantillons non amplifiés (*n* = 5) était de 3.1 log_10_ copies/mL [IQR: 3-3.6] pour une *p*-value = 0.02. Au moins une mutation conférant une résistance à une molécule antirétrovirale a été observée chez 19 patients, soit un taux de résistance globale de 14%. La mutation M184V (*n* = 13) et les TAMs (Thymidine Analogue-Associated Mutations) (*n* = 32) étaient les plus fréquemment observées pour les INTI et la K103N (*n* = 11) et Y181C (*n* = 7) pour les INNTI. Leurs survenues étaient d’autant plus marquées que la durée du traitement était élevée (Figure 2). L’insertion T69 a également été observée chez 5 patients dont un en deuxième ligne. D’autres mutations comme la V198I, G190A/G et Y188A/L ont été également notées chez 4, 3 et 2 patients respectivement (Tableau 1). Par ailleurs, aucune mutation de résistance majeure aux IP (Inhibiteur de Protéase) n’a été observée et ceci qu’il s’agisse de patients avec une virémie supérieure à 3 log_10_ copies/mL ou pas, de patients sous première ligne ou deuxième ligne de traitement.

### Phylogénie et caractérisation moléculaires des souches virale

**FIGURE 3:**
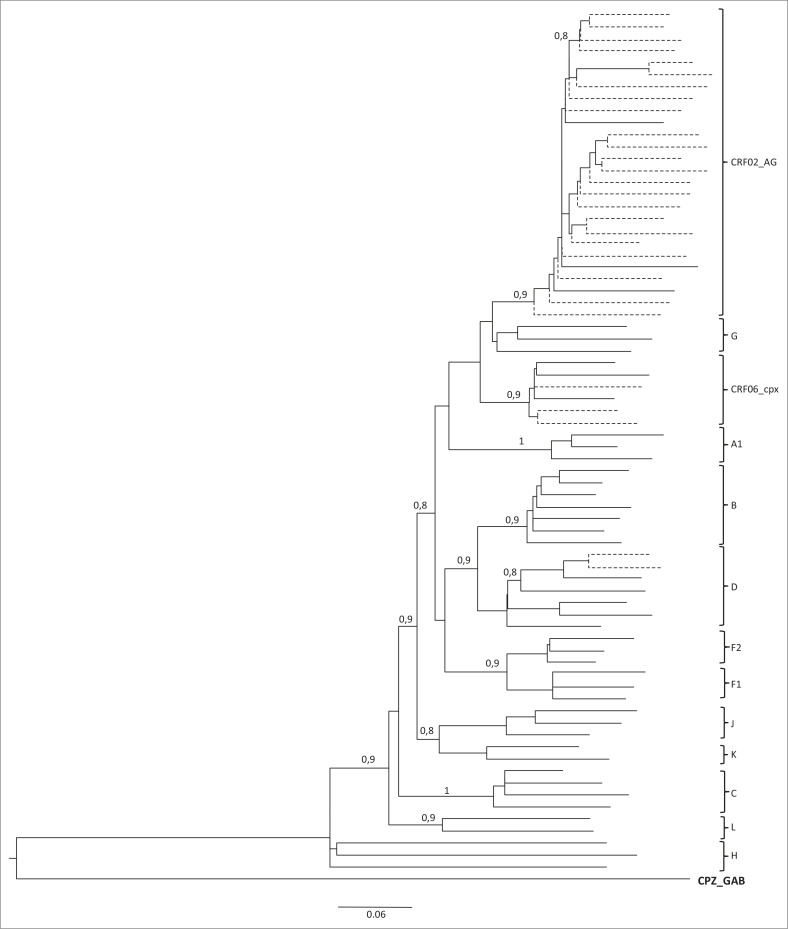
Arbre PhyML montrant la relation phylogénétique entre les séquences requêtes (*n*=28, en traits pointillés) et les références (en traits pleins) dans la région du gène pol (PR+RT) du VIH-1.

L’analyse phylogénétique des séquences nucléotides a permis de montrer la prédominance du CRF02_AG, 82% (*n* = 23/28). Les sous types D et CRF06_cpx ont été observés dans les proportions respectives 7% (*n* = 2/28) et 11% (*n* = 3/28) (Figure 3).

## Discussion

Cette étude décrit pour la première fois en Guinée les données relatives à l’échec virologique et le taux de résistance du VIH-1 chez des patients sous TARV. Précédemment, dans des conditions relativement similaires à celles décrites dans cette étude, du fait que le plasma soit le type d’échantillon de référence pour la quantification de la CV et la réalisation des tests de résistance, nous avons effectué des études d’évaluation et de faisabilité des tests virologiques à partir d’échantillons DBS. Ce fut, d’une part, des comparaisons de valeurs de CV et de profils de résistance entre échantillons pairs plasma et DBS^8^ et, d’autres part, la faisabilité des tests de résistance à partir de DBS collectés et acheminés dans des conditions réelles de vie.^10^ Le but de cette présente étude était d’évaluer la faisabilité technique et logistique des tests de CV et de génotypage à partir d’échantillons DBS de patients sous TARV collectés dans des conditions de terrain. L’échec virologique a été observé dans 24.26% (*n* = 33/136) des cas et parmi eux 2/3 des patients étaient à plus de 24 mois de traitement (Tableau 1). Le taux de suppression virologique (75.7%) semble être satisfaisant pour une médiane de suivi de 35 mois.^18^ Garrido et *al* en 2008 ont rapporté un taux de suppression virologique similaire mais avec une médiane de suivi thérapeutique de 12 mois.^7^ Dans cette étude, nous avons obtenu un taux d’amplification réussi de 84.84% comparable à ceux obtenus en Tanzanie,^19^ en Espagne^20^ et légèrement inférieur à ceux obtenus précédemment au Sénégal^10^ et en Guinée Conakry (94%).^21^ Cinq échantillons de patients en rebond virologique, dont la médiane de CV était faible (3.13 log_10_ copies/mL [3.06-3.63]), n’ont pas pu être génotypés, malgré plusieurs tentatives. Ceci pourrait également être dû à une dégradation des acides nucléiques durant les processus de conservation et de stockage des prélèvements DBS à température ambiante. D’ailleurs, plusieurs études ont rapporté un faible taux d’amplification réussi pour des échantillons à CV < 5000 (3.69 log_10_) copies/mL contrairement à ceux ayant des CV élevées (> 10000 [4 log_10_] copies/mL).^20,22,23,24^

Les mutations de résistances sélectionnées chez les patients en échec virologique dans cette étude (Tableau 1) étaient en accord avec les schémas thérapeutiques en cours ou antérieurs. La proportion de patients ayant ces mutations augmente avec la durée du traitement (14/19 ; supérieure à 24 mois). Par ailleurs, la mutation M184V conférant une résistance au 3TC/FTC et les TAMs pour les INTI et les mutations K103N et Y181C pour les INNTI (Figure 2), les plus prédominantes ici, ont été également celles observées dans plusieurs études conduites en Afrique subsaharienne.^7,25,26^ L’insertion T69, conférant une multi-résistance aux INTI^27^ et retrouvée chez certains patients, reflète la composition de leur régime thérapeutique, qui inclut soit la didanosine (ddI), soit la stavudine (d4T) ou encore la zidovudine (AZT). Ces molécules sont connues pour sélectionner la mutation T69.^28,29,30^ Par ailleurs, les résultats de génotypage ont montré que 27.2% (*n* = 9/28) des patients en échec virologique étaient porteurs de virus sauvages (*i.e.* encore sensibles aux ARV). Cet échec virologique serait probablement lié à une mauvaise observance ou encore aux variants minoritaires que le ‘bulk sequencing’ utilisé dans cette étude ne peut pas détecter. Des observations similaires ont été rapportées à Abidjan (Côte d’Ivoire) et à Bangui (République centrafricaine).^31,32^ Une virémie élevée sous TARV est associée à un risque d’émergence de la résistance du VIH aux médicaments. Ainsi, cette étude met en évidence la nécessité d’améliorer l’observance au traitement.

L’analyse phylogénétique des souches virales étudiées montre une forte prédominance du CRF02_AG (81%, *n* = 26), comme précédemment rapporté dans une étude de résistance primaire du VIH-1 chez des patients nouvellement infectés à Conakry.^21^

Ces résultats montrent la faisabilité technique et logistique des tests de CV et de génotypage à partir de prélèvements DBS. D’un autre côté, cette étude montre l’intérêt de l’utilisation des DBS comme support de prélèvement dans le monitoring virologique des patients sous TARV en Guinée, pays où le suivi virologique est encore peu structuré. De plus elle a permis de documenter le taux de patients en échec virologique, la résistance du VIH-1 aux ARV et la diversité génétique en Guinée.

## References

[1] UNAIDS. UNAIDS report on the global AIDS epidemic. 2012:pp:1–212 [UNAIDS Global AIDS Report web site]. Disponible sur: http://www.unaids.org/en/media/unaids/contentassets/documents/epidemiology/2012/gr2012JC2434_WorldAIDSday_results_en.pdf. [Consulté le 16 Mai 2013].

[2] CNLS-Guinée. Revue des progrès vers la réalisation des cibles de la déclaration 2011 de l’ONU sur le VIH et le Sida. Rapport narratif. 2014:pp:1–36. http://www.unaids.org/fr/regionscountries/countries/guinea/ [Consulté le 05 Juin 2014].

[3] CNLS-Guinée. Rapport UNGASS 2012 _Guinée. 2012:pp:1-71. [UNAIDS Global AIDS Report web site]. Disponible sur http://www.unaids.org/en/dataanalysis/knowyourresponse/countryprogressreports/2012countries/ce_GN_Narrative_Report[2011].pdf. [consulté le 26 JUIN 2013]

[4] PNPCSP-IST/SIDA. Normes et protocoles de prise en charge de l’infection par le VIH chez l’adulte et l’enfant en GUINEE. 2012:pp:1–103. [WHO web site]. Disponible sur http://www.who.int/hiv/pub/guidelines/guinea_art.pdf. [Consulté le 26 JUIN 2013]

[5] Kebe K, Ndiaye O, Ndiaye HD, et al. RNA versus DNA (NucliSENS EasyQ HIV-1 v1.2 versus Amplicor HIV-1 DNA test v1.5) for early diagnosis of HIV-1 infection in infants in Senegal. J Clin Microbiol. 2011 Jul;49(7):2590–2593. 10.1128/JCM.02402-10 21543563 PMC3147881

[6] Castro AC, Borges LG, Souza Rda S, Grudzinski M, D’Azevedo PA. Evaluation of the human immunodeficiency virus type 1 and 2 antibodies detection in dried whole blood spots (DBS) samples. Rev Inst Med Trop Sao Paulo. 2008 May-Jun;50(3):151–156. 10.1590/S0036-46652008000300004 18604415

[7] Garrido C, Zahonero N, Fernandes D, et al. Subtype variability, virological response and drug resistance assessed on dried blood spots collected from HIV patients on antiretroviral therapy in Angola. J Antimicrob Chemother. 2008 Mar;61(3): 694–698. 10.1093/jac/dkm515 18218644

[8] Kane CT, Ndiaye HD, Diallo S, et al. Quantitation of HIV-1 RNA in dried blood spots by the real-time NucliSENS EasyQ HIV-1 assay in Senegal. J Virol Methods. 2008 Mar;148(1–2):291–295. 10.1016/j.jviromet.2007.11.011 18242718

[9] Johannessen A, Garrido C, Zahonero N, Naman E, de Mendoza C. HIV-1 drug resistance testing from dried blood spots collected in rural Tanzania using the ViroSeq HIV-1 Genotyping System. J Antimicrob Chemother. 2011 Feb;66(2): 260–264. 10.1093/jac/dkq433 21115444 PMC3019084

[10] Diouara AA, Diop-Ndiaye H, Kebe-Fall K, et al. Dried blood spots for HIV-1 drug resistance genotyping in decentralized settings in Senegal. J Med Virol. 2014 Jan;86(1):45–51. 10.1002/jmv.23778 24122937

[11] Diouara AAM, Sow A, Leye N, et al. Comparaison des charges virales et des mutations de résistance entre plasma et DBS au Sénégal. Abstract Book: 6éme Conférence Francophone VIH/SIDA, 25–28 Mars - Genève Suisse. 2012;abstract numéro 82.

[12] Monleau M, Aghokeng AF, Eymard-Duvernay S, et al. Field evaluation of dried blood spots for routine HIV-1 viral load and drug resistance monitoring in patients receiving antiretroviral therapy in Africa and Asia. J Clin Microbiol. 2014 Feb;52(2):578–586. 10.1128/JCM.02860-13 24478491 PMC3911301

[13] Bertagnolio S, Parkin NT, Jordan M, Brooks J, Garcia-Lerma JG. Dried blood spots for HIV-1 drug resistance and viral load testing: A review of current knowledge and WHO efforts for global HIV drug resistance surveillance. AIDS Rev. 2010 Oct-Dec;12(4):195–208. 21179184

[14] Boom R, Sol CJ, Salimans MM, Jansen CL, Wertheim-van Dillen PM, van der Noordaa J. Rapid and simple method for purification of nucleic acids. J Clin Microbiol. 1990 Mar;28(3):495–503. 1691208 10.1128/jcm.28.3.495-503.1990 PMC269651

[15] van Deursen P, Oosterlaken T, Andre P, et al. Measuring human immunodeficiency virus type 1 RNA loads in dried blood spot specimens using NucliSENS EasyQ HIV-1 v2.0. J Clin Virol. 2010 Feb;47(2):120–125. 10.1016/j.jcv.2009.11.021 20018560

[16] Lole KS, Bollinger RC, Paranjape RS, et al. Full-length human immunodeficiency virus type 1 genomes from subtype C-infected seroconverters in India, with evidence of intersubtype recombination. J Virol. 1999 Jan;73(1):152–160. 9847317 10.1128/jvi.73.1.152-160.1999 PMC103818

[17] Gouy M, Guindon S, Gascuel O. SeaView version 4: A multiplatform graphical user interface for sequence alignment and phylogenetic tree building. Mol Biol Evol. 2010 Feb;27(2):221–224. 10.1093/molbev/msp259 19854763

[18] Barth RE, van der Loeff MF, Schuurman R, Hoepelman AI, Wensing AM. Virological follow-up of adult patients in antiretroviral treatment programmes in sub-Saharan Africa: A systematic review. Lancet Infect Dis. 2010 Mar;10(3):155–166. 10.1016/S1473-3099(09)70328-7 20185094

[19] Johannessen A, Holberg-Petersen M, Lovgaarden G, et al. HIV type-1 drug resistance testing on dried blood spots is feasible and reliable in patients who fail antiretroviral therapy in rural Tanzania. Antivir Ther. 2010;15(7):1003–1009. 10.3851/IMP1660 21041915

[20] Masciotra S, Garrido C, Youngpairoj AS, et al. High concordance between HIV-1 drug resistance genotypes generated from plasma and dried blood spots in antiretroviral-experienced patients. Aids. 2007 Nov 30;21(18):2503–2511. 10.1097/QAD.0b013e3281c618db 18025887

[21] Charpentier C, Bellecave P, Cisse M, et al. High prevalence of antiretroviral drug resistance among HIV-1-untreated patients in Guinea-Conakry and in Niger. Antivir Ther. 2011;16(3):429–433. 10.3851/IMP1754 21555827

[22] Youngpairoj AS, Masciotra S, Garrido C, Zahonero N, de Mendoza C, Garcia-Lerma JG. HIV-1 drug resistance genotyping from dried blood spots stored for 1 year at 4 degrees C. J Antimicrob Chemother. 2008 Jun;61(6):1217–1220. 10.1093/jac/dkn100 18344550 PMC2386080

[23] Rottinghaus EK, Ugbena R, Diallo K, et al. Dried blood spot specimens are a suitable alternative sample type for HIV-1 viral load measurement and drug resistance genotyping in patients receiving first-line antiretroviral therapy. Clin Infect Dis. 2012 Apr;54(8):1187–1195. 10.1093/cid/cis015 22412066 PMC11528918

[24] Monleau M, Butel C, Delaporte E, Boillot F, Peeters M. Effect of storage conditions of dried plasma and blood spots on HIV-1 RNA quantification and PCR amplification for drug resistance genotyping. J Antimicrob Chemother. 2010 Aug;65(8):1562–1566. 10.1093/jac/dkq205 20542904

[25] Liegeois F, Vella C, Eymard-Duvernay S, et al. Virological failure rates and HIV-1 drug resistance patterns in patients on first-line antiretroviral treatment in semirural and rural Gabon. J Int AIDS Soc. 2012;15(2):17985. 10.7448/IAS.15.2.17985 23199801 PMC3510650

[26] Dagnra AY, Vidal N, Mensah A, et al. High prevalence of HIV-1 drug resistance among patients on first-line antiretroviral treatment in Lome, Togo. J Int AIDS Soc. 2011;14:30. 10.1186/1758-2652-14-30 PMC3125306 21663632

[27] Johnson VA, Calvez V, Gunthard HF, et al. 2011 update of the drug resistance mutations in HIV-1. Top Antivir Med. 2011 Nov;19(4):156–164. 22156218 PMC6148877

[28] De Antoni A, Foli A, Lisziewicz J, Lori F. Mutations in the pol gene of human immunodeficiency virus type 1 in infected patients receiving didanosine and hydroxyurea combination therapy. J Infect Dis. 1997 Oct;176(4):899–903. 10.1086/516511 9333147

[29] Winters MA, Coolley KL, Girard YA, et al. A 6-basepair insert in the reverse transcriptase gene of human immunodeficiency virus type 1 confers resistance to multiple nucleoside inhibitors. J Clin Invest. 1998 Nov 15;102(10):1769–1775. 10.1172/JCI4948 9819361 PMC509125

[30] Scherrer AU, von Wyl V, Joos B, et al. Predictors for the emergence of the 2 multi-nucleoside/nucleotide resistance mutations 69 insertion and Q151M and their impact on clinical outcome in the Swiss HIV cohort study. J Infect Dis. 2011 Mar 15;203(6):791–797. 10.1093/infdis/jiq130 21285456 PMC3119329

[31] Messou E, Chaix ML, Gabillard D, et al. Increasing rate of TAMs and etravirine resistance in HIV-1-infected adults between 12 and 24 months of treatment: The VOLTART cohort study in Cote d’Ivoire, West Africa. J Acquir Immune Defic Syndr. 2013 Jun 21. 10.1097/QAI.0b013e3182a009e4 PMC3834582 23797690

[32] Pere H, Charpentier C, Mbelesso P, et al. Virological response and resistance profiles after 24 months of first-line antiretroviral treatment in adults living in Bangui, Central African Republic. AIDS Res Hum Retroviruses. 2012 Apr;28(4): 315–323. 10.1089/aid.2011.0127 21942692

